# Activated Carbon Fibers with Hierarchical Nanostructure Derived from Waste Cotton Gloves as High-Performance Electrodes for Supercapacitors

**DOI:** 10.1186/s11671-017-2151-4

**Published:** 2017-06-02

**Authors:** Chao Wei, Jianlin Yu, Xiaoqing Yang, Guoqing Zhang

**Affiliations:** School of Materials and Energy, Guangdong University of Technology, Guangzhou, 510006 People’s Republic of China

**Keywords:** Supercapacitors, Porous carbon materials, Carbon fiber, Activation, Hierarchical nanostructure

## Abstract

One of the most challenging issues that restrict the biomass/waste-based nanocarbons in supercapacitor application is the poor structural inheritability during the activating process. Herein, we prepare a class of activated carbon fibers by carefully selecting waste cotton glove (CG) as the precursor, which mainly consists of cellulose fibers that can be transformed to carbon along with good inheritability of their fiber morphology upon activation. As prepared, the CG-based activated carbon fiber (CGACF) demonstrates a surface area of 1435 m^2^ g^−1^ contributed by micropores of 1.3 nm and small mesopores of 2.7 nm, while the fiber morphology can be well inherited from the CG with 3D interconnected frameworks created on the fiber surface. This hierarchically porous structure and well-retained fiber-like skeleton can simultaneously minimize the diffusion/transfer resistance of the electrolyte and electron, respectively, and maximize the surface area utilization for charge accumulation. Consequently, CGACF presents a higher specific capacitance of 218 F g^−1^ and an excellent high-rate performance as compared to commercial activated carbon.

## Background

Porous carbon material (PCM)-based supercapacitors have triggered increasing interest during the past decades by virtue of their high power density, fast charge-discharge rate, and long cycling stability [1–10]. They have been widely used as power sources for versatile applications requiring quick bursts of energy, such as high-power electronic devices, electric vehicles (EVs), and hybrid EVs [11, 12]. It is well known that the supercapacitive performance of PCMs strongly depends on their nanostructure. Although clear efforts aimed at designing advanced PCMs with well-defined nanostructure for optimizing supercapacitive properties are hot research aspects, e.g., carbon nanospheres [13, 14], carbon nanotubes [15, 16], and carbon nanorods [17, 18], their practical applications are significantly limited by the high cost, multi-step processes, and heavily usage of toxic strong oxidants [19].

Hitherto, with the decreasing availability of fossil-based PCMs, activated PCMs (APCMs) derived from biomass/waste materials of coconut shells and woods are still the commercial choice for supercapacitor electrodes because of their high performance-cost ratio and simple preparation process [20–24]. Numerous efforts have also been devoted to exploring novel biomass/waste-based APCMs for further improving their supercapacitive performance, such as cigarette filter, cigarette ash, tea leaves, human hair, and fish scale [25–28] Nevertheless, although a relatively large surface area can be obtained for electric double-layer formation during the activating process, such APCMs are usually lack of meso/macroporosity for electrolyte diffusion/transfer owing to the bulk nanostructure of the biomass/waste precursors and/or the poor structural inheritability during the activating process. This relatively low electrolyte diffusion/transfer efficiency usually results in low surface area utilization, particularly under high current densities. For example, Wang et al. prepared a kind of chicken feather-based APCMs by KOH activation. It possessed a low capacitance retention of 55% as the current density was increased from 1 to 10 A g^−1^ owing to its micropore-dominant structure [29]. Another class of willow leaf-based APCMs was obtained by Liu et al. through ZnCl_2_ activation. It also displayed a poor capacitance retention of 70% while increasing the current density from 1 to 5 A g^−1^, because its original nanostructure was completely destroyed during the activating process [30]. Thus, carefully selecting a suitable biomass/waste precursor with developed nanostructure and good structural inheritability during the activating process is highly recommended but remains challenging.

**Fig. 1:**
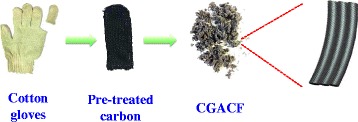
Schematic diagram for the preparation of CGACF

In the present work, we prepare a class of activated carbon fibers by selecting waste cotton gloves (CG) as the precursor (Fig. 1). CG, a readily recycled waste product generated in daily life, is usually abandoned as garbage. It mainly consists of cellulose fibers that can be transformed to carbon along with good inheritability of the fiber morphology upon pyrolysis/activation. As prepared, the CG-based activated carbon fiber (CGACF) demonstrates a surface area of 1435 m^2^ g^−1^ donated by micropores of 1.3 nm and small mesopores of 2.7 nm, while the fiber morphology (several microns in diameter) can be well inherited from the CG with a 3D interconnected frameworks created on the fiber surface. This hierarchical porous structure and well-retained fiber-like skeleton can simultaneously minimize the diffusion/transfer resistance of the electrolyte and electron, respectively, and maximize the surface area utilization for charge accumulation. Consequently, CGACF presents a higher specific capacitance of 218 F g^−1^ and more excellent high-rate performance as compared to commercial activated carbon (AC).

## Experimental

### Preparation of CGACF

CGACF was prepared through a chemical activating route using KOH and recycled waste CG fibers as the activating agent and carbon precursors, respectively. During a typical procedure, CG fibers were cut into millimeter-scale debris and then pre-carbonized at 500 °C for 3 h under nitrogen atmosphere. Subsequently, the pre-carbonized CG (PCCG) was mixed with KOH with a KOH/PCCG mass ratio of 1.5 and then carbonized at 900 °C with a heating rate of 5 °C min^−1^ for 3 h under nitrogen atmosphere. The resulting activated product (CGACF) was washed repeatedly with acid and distilled water until the pH value of the filtrate reached 7. After that, CGACF was dried at 80 °C for 12 h. Meanwhile, another sample of non-activated carbon fiber was prepared through the same procedure without adding KOH and denoted as CGCF. For comparison, a commercially available AC for supercapacitors was purchased from Kuraray Chemical Co., Ltd., and served as a reference.

### Structure Characterization

X-ray diffraction (XRD) patterns were recorded on a D/MAX 2200 VPC equipment using Kα radiation as an X-ray source. Raman spectra were used to confirm the graphitized feature by a Renishaw inVia 2000 spectrometer. The morphology and nanostructure of the samples were observed by a scanning electron microscope (SEM; JSM-6330F) and a transmission electron microscope (TEM; JEOL JEM-2010). Nitrogen adsorption-desorption isotherms were measured at 77 K on a Micrometrics ASAP 2460 surface area and porosity analyzer. Brunauer-Emmett-Teller (BET) method was utilized to calculate the BET surface area (*S*_BET_). The micropore volume (*V*_mic_), micropore surface area (*S*_mic_), mesopore volume (*V*_mes_), mesopore surface area (*S*_mes_), and pore size distribution (PSD) curves of the samples were analyzed by t-plot, Barrett-Joyner-Halendar, and density functional theory (DFT), respectively.

### Electrochemical Measurements

The electrochemical performances of the materials were conducted in 1 M H_2_SO_4_ using a sandwich-type two-electrode testing cell. To prepare the working electrode, the active material was mixed with carbon black and poly (vinylidene difluoride) (PVDF) at a mass ratio of 8:1:1. The obtained paste was pressed onto the current collector of titanium foil uniformly under 10 Mpa and dried in vacuum at 120 °C for 12 h. Galvanostatic charge-discharge (GCD) tests were performed using a Neware battery test equipment (CT2001A) at current densities from 0.1 to 20 A g^−1^. Cyclic voltammetry (CV) at a scan rate of 200 mV s^−1^ and electrochemical impedance spectroscopy (excitation signal: 5 mV and frequency range 0.001–100,000 Hz) were also recorded using an IM6ex electrochemical workstation. The specific capacitance *C*_g_ (in F g^−1^) of the samples was calculated from the discharge curves by the formula of ${C}_{\mathrm{g}}=\frac{I\cdot \varDelta t}{\varDelta U}\cdot \frac{m_1+{m}_2}{m_1\cdot {m}_2}$, where *I* was the discharge current (A), △*t* was the discharge time (s), △*U* was the potential change during the discharge process (V), *m*_1_ and *m*_2_ were the mass of the active materials in the electrodes (g).

## Results and Discussions

**Fig. 2:**
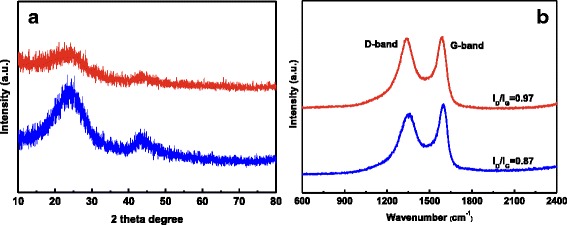
XRD patterns and Raman spectra. **a** XRD patterns and **b** Raman spectra of CGCF (*blue line*) and CGACF (*red line*)

The XRD patterns of the CGCF and CGACF samples in Fig. 2a both possess two relatively broader peaks around 23.5° and 44° (2*θ*), which correspond to the (002) and (100) diffraction of hexagonal graphite, respectively [31, 32]. The decreased intensity of the diffraction peak with KOH activation is ascribed to the turbostratic carbon structure with randomly oriented graphene layers in CGACF, implying a much more developed porosity of CGACF compared with that of CGCF [33]. Raman spectra of the samples are displayed in Fig. 2b. The peak located at about 1350 cm^−1^ is assigned to the D-band, which should be related to the sp^3^ carbon atoms of disordered or defective carbon. The peak at about 1590 cm^−1^ is referred to the G-band, corresponding to the fingerprint of graphitic crystallites of carbon [34]. The higher relative intensity ratio (*I*_D_/*I*_G_) of CGACF, in comparison to that of CGCF, confirms the much more developed porosity, i.e., structural defects.

**Fig. 3:**
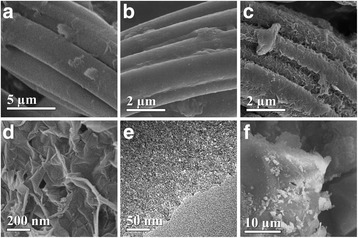
SEM and TEM images. SEM images of **a** raw CG, **b** CGCF, **c**, **d** CGACF, and **f** commercial AC. **e** TEM image of CGACF

Figure 3 shows the SEM and TEM images of the AC, CGCF and CGACF samples. In Fig. 3a, raw CG displays a fiber-like morphology with ca. 3 μm in diameter and a smooth surface of the fiber wall. After carbonization without KOH, the diameter of the fiber shrinks to about 2 μm due to the emission of many non-carbon elements and some carbon-containing compounds during carbonization, while the fiber surface remains smooth (Fig. 3b). Retaining the well-defined morphology of the precursor in the activating procedure is critical to the preparation of an advanced PCM with large surface area for charge accumulation and effective electrolyte/electron diffusion/transfer pathways. It is fortunate that the fibrous structure of CGACF is well retained after the activating process, and a rough surface morphology constructed by 3D interconnected frameworks is obtained derived from the intensive etching effect of KOH (Fig. 3c, d). These developed surface frameworks, which stack into numerous macropores, and well-retained fiber structure are very important for the supercapacitors application, since they can provide high-efficient electrolyte diffusion/transfer pathways and conductive skeleton, respectively. In addition, the TEM image of CGACF reveals the existence of abundant small nanopores on the surface of the fiber (Fig. 3e). In a sharp contrast, large carbon bulks without any regular and/or hierarchically porous structure are observed in commercial AC (Fig. 3f).

**Fig. 4:**
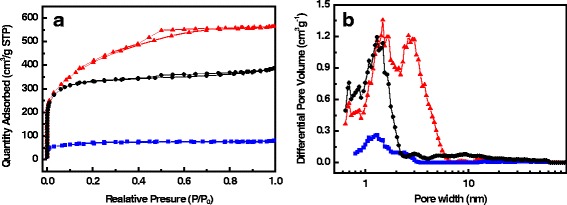
Nitrogen adsorption-desorption tests. **a** Nitrogen adsorption-desorption isotherms at 77 K and **b** their corresponding PSD curves of the CGACF (*red line* and *symbol*), CGCF (*blue line* and *symbol*) and AC (*black line* and *symbol*) samples

N_2_ adsorption-desorption isotherms were utilized to analyze the nanostructure of the samples quantitatively. As shown in Fig. 4a, the CGACF sample presents a much higher adsorption uptake at low relative pressure (*P*/*P*_0_) compared with CGCF, implying that the microporosity of the fibrous structure is significantly increased due to the activation treatment. In addition, the obvious hysteresis loop at the medium *P*/*P*_0_ indicates the formation of many small mesopores in CGACF. According to the DFT PSD curve of CGACF in Fig. 4b, numerous micropores and small mesopores are concentrated at 1.3 and 2.7 nm, respectively, consisting with the TEM observation. It is believed that the KOH activation not only creates a substantial number of micropores but also continues widening them into small mesopores [21, 35, 36]. Comparatively, the commercial AC only shows an uptake at low *P*/*P*_0_, suggesting a micropore peak at 1.3 nm (Fig. 4b) and a micropore-dominant surface area of 1282 m^2^ g^−1^ (primarily contributed by *S*_mic_, Table 1).

**Table 1:** Pore parameters of the samples

Sample	*S*_BET_ (m^2^ g^−1^)	*S*_mic_ (m^2^ g^−1^)	*S*_mes_ (m^2^ g^−1^)	*V*_total_ (cm^3^ g^−1^)	*V*_mic_ (cm^3^ g^−1^)	*V*_mes_ (cm^3^ g^−1^)
CGCF	252	239	49	0.12	0.10	0.04
CGACF	1435	852	839	0.87	0.42	0.58
AC	1282	1192	156	0.58	0.48	0.15

We prospect that this integration of the large surface area provided by the hierarchically micro/mesoporous structure, 3D interconnected frameworks on the fiber surface and well-retained fiber skeleton will be beneficial for the supercapacitive behaviors. Thus, various electrochemical measurements were carried out using the assembled sandwich-type two-electrode testing cells. CV measurement and EIS tests were first performed to evaluate the electrolyte/electron migrating capability.

**Fig. 5:**
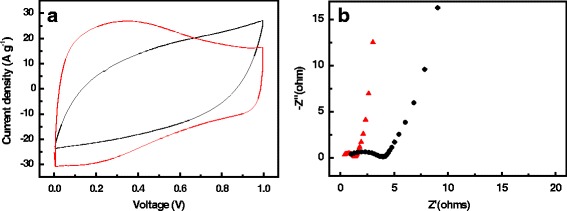
CV measurements and EIS tests. **a** CV curves at the scan rate of 200 mV s^−1^ and **b** Nyquist plots of the CGACF (*red line* and symbol) and AC samples (*black line* and symbol)

Commonly, the rectangular shape of the CV curves at a relatively high scan rate (usually 20–200 mV s^−1^) can be used to estimate the ability of ion diffusion/transfer within the nanocarbon structure [37–40]. Apparently, the CV curve of CGACF at 200 mV s^−1^ shows a near-rectangular shape, as compared to that of AC with a distorted shape (Fig. 5a), indicating the effective electrolyte accessibility and fast ion transportation in the nanostructure of CGACF. Nyquist plots obtained from the EIS tests also confirm this assumption (Fig. 5b). As we know, the initial intersection between the curve and Z’ axis reflects the equivalent series resistance (ESR) of the electrode, while the diameter of the semicircle in the high-frequency region reflects the polarization resistance or charge transfer resistance (*R*_p_/*R*_ct_) [41]. CGACF presents much lower *R*_p_/*R*_ct_ and ESR of 0.94 and 0.42 Ω compared to AC-YP (2.90 and 1.03 Ω, respectively). These aforementioned results reveal the excellent electrolyte/electron migrating capability of CGACF derived from the hierarchically porous structure and good inheritance of the fiber skeleton.

**Fig. 6:**
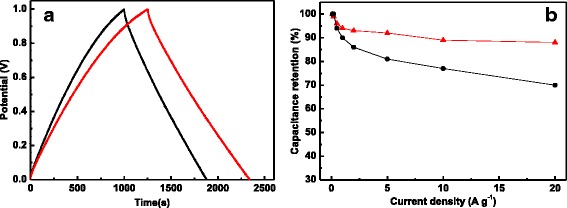
GCD tests and capacitance retention. **a** GCD curves at the current density of 0.1 A g^−1^ and **b** capacitance retention at various current densities of the CGACF (*red line* and *symbol*) and AC samples (*black line* and *symbol*)

Based upon these unique nanostructure characteristics, CGACF demonstrates the highest *C*_g_ in the GCD tests and an excellent capacitance retention under high charge-discharge rates (Fig. 6a, b). As calculated from the discharge time, the *C*_g_ of CGACF and commercial AC is 218 and 175 F g^−1^ at the current density of 0.1 A g^−1^, respectively. Incredibly, as raising the current density to an extremely high value of 20 A g^−1^, a high capacitance retention of 88% (192 F g^−1^) is obtained, whereas that of AC is deceased sharply to 70%.

**Fig. 7:**
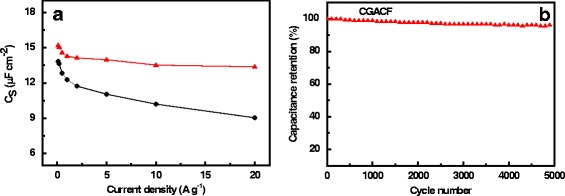
*C*_S_ and cycling stability tests. **a** *C*_S_ at different current densities and (**b**) cycling stability at the current density of 1 A g^−1^ for 5000 cycles of the samples. CGACF: *red line* and *symbol*; AC: *black line* and *symbol*

Another dominant characteristic to represent the mass diffusion/transfer capability is the efficient ion-accessible surface area, which can be appraised by the capacitance per surface area (*C*_S_). Generally, a high *C*_S_ represents a high surface area utilization. Increasing the charge-discharge rate usually leads to a sharp decrease of the *C*_S_ due to the insufficient time of ion diffusion and charge arrangement. Obviously, both the CGACF and commercial AC samples show similar *C*_S_ of 13–15 μF cm^–2^ at 0.1 A g^–1^ (Fig. 7a), implying their comparable ion accessibility of the surface area at such a low current density. Nevertheless, as increasing the charge-discharge rate, AC shows a much steeper decreasing trend in comparison with CGACF. For example, a low *C*_S_ of 9 μF cm^−2^ is obtained at 20 A g^−1^ for AC, whereas the *C*_S_ of CGACF stays above 13 μF cm^−2^. This value, to our knowledge, is much better than that of most biomass/waste-based APCMs at high current density [42–46]. In addition, after repeating the charge-discharge tests for 5000 cycles at a current density of 1 A g^−1^, CGACF exhibits a good cycling durability with a capacitance retention of 96.3% (Fig. 7b).

Overall, the excellent supercapacitive performance of the CGACF including high *C*_g_, cycling stability, and superior high-rate capability can be ascribed to the following factors: (1) the high surface area of 1435 m^2^ g^−1^ contributed by the hierarchical meso/micropores provides many active sites for energy storage; (2) the 3D interconnected frameworks on the fiber surface coupled with the well-retained fiber morphology offer high-efficient electrolyte and electron diffusion/transfer pathways for ensuring a high utilization of the surface area and outstanding conductive skeleton, respectively, especially under high current densities.

## Conclusions

A new class of activated carbon fibers with hierarchical nanostructure derived from waste CG is successfully fabricated. Based on the good inheritance of fiber morphology in CG and etching effect of KOH, the obtained CGACF demonstrates a high specific surface area of 1435 m^2^ g^−1^ donated by micropores of 1.3 nm and small mesopores of 2.7 nm, while the fiber-like morphology can be well inherited from the CG with a 3D interconnected frameworks created on the fiber surface. Consequently, CGACF shows a much higher *C*_g_ of 218 F g^−1^ at 0.1 A g^−1^ and excellent high-rate capability (88% at 20 A g^−1^) compared with commercial AC (175 F g^−1^ and 70%, respectively). Furthermore, CGACF exhibits a good cycling durability with a capacitance retention of 96.3% at the current density of 1 A g^−1^ after 5000 cycles. We hope that this study would open up new opportunities in the development of biomass/waste-based APCMs for high-performance energy storage devices.
